# Signalling Standards for Progress: Bridging the Divide Between a Valid Consent to Use Patient Data Under Data Protection Law and the Common Law Duty of Confidentiality

**DOI:** 10.1093/medlaw/fwab014

**Published:** 2021-07-16

**Authors:** Edward S Dove, Mark J Taylor

**Affiliations:** 1 School of Law, University of Edinburgh, UK; 2 Melbourne Law School, University of Melbourne, Australia

**Keywords:** confidentiality, consent, data protection, health research, medicine, patient data

## Abstract

In this article, we analyse the legal components of disclosing confidential patient information under the UK’s common law duty of confidentiality (CLDoC) and processing personal (health) data under the UK’s General Data Protection Regulation (GDPR) and Data Protection Act 2018. We describe the ostensible divide between the CLDoC and data protection law when it comes to the requirements of a valid *signal* of consent by a patient to use and disclose patient information, obtained by a health professional in the context of direct care, for health care and health research purposes. Ultimately, our analysis suggests that we are saddled, at least in the medium term, with two regimes operating with different standards of a valid consent—while putatively protecting similar interests. There is, however, opportunity for progress. It is possible to improve professional guidance on the interaction between the regimes and to achieve significant *normative* alignment without aligning the signalling standard for consent; this would promote consistent protection of reasonable expectations of patients across both regimes. Further coherence would require aligning not only the standard, but also the role played by consent under each regime. Here we argue that, in relation to direct care, any such shift should be away from consent as the normal justification. In relation to health research, on the contrary, it should be toward consent as the normal justification for use and disclosure of patient information under both the CLDoC and data protection law.

## I. INTRODUCTION

On 2 October 2019, the National Data Guardian (NDG), an independent, non-regulatory, advice-giving body in England sponsored by the Department of Health and Social Care,^1^ tweeted a series of key points emerging from the ‘Healthcare Excellence Through Technology’ conference happening in London. One tweet highlighted a discussion between Dame Fiona Caldicott, Prof Andrew Morris, and patient advocate Paul Charlton, and noted that: ‘NDG on GDPR and common law duty of confidentiality: guidance is needed to help frontline clinicians understand interplay between the two. NDG working with @ICO to look at what can be done to help the system with this.’^2^ This sparked a discussion between the two of us, given our long-standing interest in both data protection law and confidentiality, as to how we might contribute to this guidance. It is an area that we agree lacks a fair amount of clarity.

A particularly problematic aspect of this is that, in the UK, health professionals (including both ‘frontline clinicians’ noted by the NDG, as well as those who engage in clinical practice and health research, such as clinical geneticists and clinicians involved in biobanks) receive mixed messages regarding what is sufficient, from a legal perspective, to signal a valid consent to use and disclose a patient’s health information.^3^ This is principally because of a lack of clarity and consistency in professional guidance regarding the conditions sufficient to *imply* a patient’s consent to use confidential information under the Common Law Duty of Confidentiality (CLDoC).^4^ Specifically, the necessity for an unambiguous signal from a patient to use their health information is, rather ironically, ambiguously signalled in guidance. This is significant given implied consent^5^ is routinely relied upon by the health (and social care) systems across the UK to provide care and conduct local audits. There is greater consistency, but still not absolute clarity, in guidance on the requirements for consent (under the CLDoC) for uses beyond care or local audit, such as health research. The clearest and most consistent statements on the requirements of an active and unambiguous signal of consent are found not in the common law, but in a statutory legal regime, namely data protection law.^6^ This is not as helpful in health care and research as it might be in other contexts, because health professionals and researchers are advised normally *not* to seek a patient’s consent simply for the purposes of meeting obligations under data protection law.^7^

It is important for the UK’s national health systems, and those working in it, to address the NDG’s call and provide greater clarity regarding how, if at all, the CLDoC and data protection law work together. To date, there is no professional guidance making clear how the evidentiary requirements for a valid signal of consent align and interact under the two regimes.^8^ We need answers to questions such as the following: can a valid consent under the CLDoC ever meet the requirements of a valid consent under data protection law? If consent does meet the relevant standard required, then is it irrelevant from a data protection perspective if sought to comply with the CLDoC? What are the consequences for data protection law if a patient’s consent is withheld or withdrawn? These questions have not been satisfactorily addressed or answered in the literature.^9^ Yet, given that much of the edifice of the UK’s health and social care enterprise relies on lawful uses of patient and citizen data, the consistency of the requirements of a valid consent under two regimes, and the interaction between them, must be rigorously explored.

In this article, we take up the NDG’s call and consider how the legal regimes of the CLDoC and data protection might align and interact. We ask whether, and how, any lack of alignment in law, or clarity in commentary, might be addressed in the medium to long term. We suggest, following our extended analysis, that there is rather limited opportunity in the medium term for the formal requirements of a valid consent to be deemed adequately similar between the two regimes such that they can be fully ‘bridged’, but there are opportunities for progress—a partial bridging—and improved clarity in guidance on both alignment and interaction.

The CLDoC and data protection law each have quite different histories, and consent requirements are situated within different legal traditions. To fashion a regulatory approach able to see the regimes operating optimally together—if not intertwined, then functioning in normatively synchronous and harmonious parallel—we must first understand how each operates currently in its own context. In other words, we must first look to see the extent to which there is conceptual alignment between signalling standards before establishing whether there might be normative alignment. While clearing the ground, we see where (and why) professional guidance is currently unclear on what constitutes a valid signal of consent for the purposes of the CLDoC. From there, we proceed to recommend intelligent and progressive alignment, moving gradually toward not only more normatively aligned consent requirements, but complete alignment between the signalling standards.

Partial conceptual (or definitional) alignment can be achieved by drawing together the requirements of an ‘implied consent’ under the CLDoC and ‘consent’ for processing personal data under General Data Protection Regulation (GDPR) Article 6(1)(a). A gulf would remain between what is sufficient for a valid consent under the CLDoC and an *explicit* consent for processing special category health data under GDPR Article 9(2)(a); however, it is still possible for the regimes to operate in more harmonious parallel: significant normative alignment between CLDoC and data protection law can be achieved *without* more fully aligning the signalling standard conceptually. Understanding how the consent requirements are nested within respective regulatory contexts, within distinct matrices of complementary safeguard, helps to illuminate how they could better normatively align and interrelate harmoniously.

In the long term, we argue in this article that the advantages of more fully aligning the signal standard cannot be achieved without shifting the role of consent under each regime. While substantive alignment, across both the normative and conceptual axes, would improve conceptual clarity, it would require reconsidering the role of consent as ‘normal’ justification under the CLDoC and ‘extraordinary’ justification under data protection law. In relation to direct care,^10^ it would require a pivot away from relying upon consent as the normal justification under the CLDoC. In relation to research, it would require a pivot toward consent as the normal justification under data protection law.^11^ More completely aligning signal standards in this way would provide a coherence and clarity to the law that is currently lacking.

Our analysis has three parts. In Section II, we describe an abstract spectrum of possibility vis-à-vis consent signalling requirements that may apply equally across both legal regimes. We outline three ideal-typical levels of increasingly stringent consent signalling requirements. Consideration of each is enriched by reflection on the functions of consent in a health care context. We posit different functions of consent and consider the implications for the evidentiary requirements of a valid signal. This abstract spectrum provides a common analytical framework with which to analyse the two legal regimes. This will prove crucial to intelligent progress towards the regimes working together coherently.

In Section III, we move to consider how and why health professionals are currently receiving mixed messaging regarding the legal requirements for consent. We describe when and what kind of consent is required by data protection law and the CLDoC, and reflect on some of the practical implications. In this section, we discuss the significance of the concepts of ‘confidential patient information’ and ‘personal data’ (and ‘data concerning health’ as a subset of personal data) and the extent of overlap between them. Then, we consider the requirements of consent under both regimes and map the concepts, and current guidance, against our framework and the three ideal-types of consent signalling requirements. This makes clearer the differences and highlights inconsistency in professional guidance, especially with regard to the validity and evidentiary requirements of a consent that is implied by conduct, but not expressed through oral or written statement. It also makes clearer the opportunities for progress by clarifying through guidance where signalling standards do align, but recognises such clarification may have little practical effect. More significantly, it opens the way for us to explore the interaction between the two regimes and consider the consequences, if any, for responsibilities under data protection law if consent is obtained to satisfy a common law duty. We conclude this section by indicating opportunities for normative synchronicity even while concepts are not fully aligned conceptually. The requirements for normative synchronicity can, however, only be achieved if consent requirements are understood within their respective regulatory contexts.

Finally, in Section IV, we consider whether there would be any advantage from a legal perspective to moving from an unsatisfactory status quo to a situation where the requirements of a valid consent are more fully bridged. We argue that any further progress regarding alignment of signalling standards (across normative *and conceptual* axes) is dependent on a more fundamental shift in the role of consent in both regimes. We argue most significant progress is reliant upon two significant changes: (i) a move away from consent as the normal justification for the use and disclosure of patient information under the CLDoC for the purposes of direct care and local clinical audit, and (ii) a move towards consent as the normal justification for disclosure by a health professional for research purposes under data protection law.^12^ Normative alignment can be achieved to some extent without full conceptual alignment; however, full conceptual (or definitional) alignment *and* normative alignment—a comprehensive bridging—is dependent upon fundamentally reconsidering the role of consent under each regime.

## II. CONSTRUCTING AN ANALYTICAL FRAMEWORK FOR ANALYSIS

Our aim in Section II is to develop a common analytical framework that will help make sense of the requirements for a valid signal of consent under *either* the CLDoC or data protection law; this framework will then be applied in our analysis in Sections III and IV. We want to do two things at this preliminary stage. The first is to re-consider the possible value and function of consent, especially in the health care and research context. The second is to identify three relatively distinct points on a spectrum of increasingly stringent signalling requirements. The first exercise gives the second significance: clarity about the value and function of consent enlivens the identification of the significance of different signalling requirements. If we can be clear on these aspects from the outset, then it will help us when we consider how and why it might be sensible, or not, to seek alignment across the two legal regimes.

This framework is an avowedly theoretical construct. We make no claims regarding its grounding in current law, guidance, or practice. Our claim is only that it will prove valuable when explaining how professional guidance on the signalling standards of the CLDoC and data protection law are different, why that matters, and what we might do about it. We begin by positing that consent might be said to do a number of things in the context of the use and disclosure of patient information/personal health data.

### A. The Functions and Dimensions of Consent

Consent may be seen as holding several functions. Within the contexts of health and the CLDoC and data protection law, we identify two core functions that may be at play. First, consent may function as a *safeguard*.^13^ Disclosure of personal data invariably carries some risk and personal health data can be seen to carry a higher level of risk than other kinds of data.^14^ The safeguarding function of consent respects a person as a competent, autonomous agent who ought not to be instrumentalised; there is a rebuttable presumption that people’s data ought not to be used in ways that expose them to risks they have not accepted. As competent, autonomous agents, individuals are entitled to voluntarily assume the risk of data disclosure and use. Consent acts as a safeguard because it averts a wrong.^15^

Secondly, consent may function as an *enabler*.^16^ The enabling function respects and, as the word indicates, enables a person’s autonomy. This may go beyond preserving an individual’s capacity for action and positively extend it. As competent, autonomous agents, people are entitled to use ‘their’ data to pursue self-selected ends. Choosing whether to disclose one’s personal health data so that others can use it, in ways that would not be lawful without consent, is an exercise of self-determination.

Both functions presume consent to be an ongoing process during which an individual can change their mind. Ascribing consent with both functions also presumes that an individual is able to understand how to safeguard their interests and function as a competent, autonomous agent. This may not always be the case (eg in relation to a very young child). It also ignores other factors, such as public interest considerations, which certainly enter the decision-making domain in health and social care.^17^ We note that the perspective we are outlining is—for now at least—determinedly individualistic. Notwithstanding the above qualification, this autonomy-focused perspective is offered as a principal consideration in justifying why consent is important.

Accepting that consent may serve both a safeguarding and an enabling function does not imply that the relationship between them is always harmonious. Indeed, it is important to recognise the independence of the functions: people can choose to be unsafe, and people can be protected by measures that they do not choose. Although ‘safeguarding’ and ‘enabling’ may not always pull in the same direction, they may, nevertheless, be yoked to the same cart: consent can be understood to be required to perform both functions in the circumstances of data processing for purposes of health care or health research.

In what follows, we assume standards of a valid consent are calibrated with both functions in mind. This does not mean that it will always be clear that each is attributed equal value. In fact, we will suggest that it may be due, at least in part, to differences of emphasis between safeguarding and enabling that variations in signalling standard arise. For example, a high evidentiary standard (eg independently witnessed signature) may be overprotective from the perspective of self-determination (if timely compliance is impracticable).

It is relatively uncontroversial to assert that for consent to be ethically and legally valid, it must be appropriately informed, freely given, and signalled by someone with capacity. We suggest that *each of these requirements* may be interpreted differently according to how emphasis is distributed between these two functions. For example, any imbalance in power might jeopardise the ‘freedom’ of a consent if the emphasis is on safeguarding; the very fact of power imbalance might be considered sub-optimal and taint the conditions for a valid consent. However, if one places emphasis upon enabling rather than safeguarding, then some imbalance in power might be benign or even desirable—if a more powerful partner helps to achieve objectives the individual could not achieve alone.

We focus on the relationship between these functions for how consent needs to be *signalled* by the person providing it to a health care professional. This is because there is currently a lack of clarity in guidance (as noted above), and putative inconsistency in the legal requirement, in relation to the evidentiary requirements of valid signal of consent (a ‘signalling standard’) in this context under the CLDoC and data protection law. The question of what constitutes an appropriate ‘signalling standard’ is to be assessed in light of the perceived need for consent to serve these functions, informed by a view on whether any safeguarding or enabling need is otherwise met or overridden by other considerations in the circumstances.

### B. Three Ideal-Types of Signalling Requirements in the Health Context

There are infinite possible variations in what could be understood to be a sufficient signal of consent to use or disclose personal health data in the health context. For the sake of our analysis, we need to identify three ideal-typical kinds of behaviour. These correspond with increasingly demanding signalling standards. Each can be described relative to the need to assure a safeguarding and/or enabling function in the circumstances. In each case, consent is necessary because use or disclosure without it would be considered a legal wrong; consent thus enables action that would otherwise be unlawful. These ideal-types can be labelled 1–Passive (implied); 2–Active (implied); and 3–Explicit.^18^

**Level 1**: Consent is *passively implied* by general conduct; here, the signal is ambiguous but there is no evidence of dissent. Level 1 may be considered sufficient to safeguard interests where risks are low or otherwise mitigated. It may respect autonomy where there is independent reason to think that the individual accepts the use or disclosure is appropriate and there are accessible means to signal objection/no consent to the use or disclosure. Phrased another way, silence or inaction in the circumstances may be sufficient. Level 1 can be adopted in what is sometimes described as an ‘opt-out’ consent model.^19^ A higher evidentiary threshold for consent is not justified by the (otherwise mitigated) risks and might disproportionately interfere with autonomy.^20^

**Level 2**: Consent is *actively implied* by deliberate act; that is, consent is *unambiguously signalled* by the person’s conduct. This may be considered sufficient where the risks are non-trivial (not otherwise substantively mitigated) and the act provides adequate reason to think that the use or disclosure itself is wanted. Silence or inaction is not enough, but any kind of conduct (eg nod of head or completion and return of questionnaire) may be sufficient if it constitutes an unambiguous signal in the circumstances.^21^

**Level 3**: Consent is *signalled explicitly by (written or oral) statement*. This may be considered necessary where risks are significant (and otherwise unmitigated) or there is reason to doubt that the use or disclosure enabled by consent is wanted by the individual. At this level, the risk of undermining the enabling (autonomy-promoting) function by requiring an express statement is justified by the significance of the risk and the doubt about wants.^22^

Consent is not always going to be required to *use or disclose* patient information in a health context.^23^ There are situations where disclosure without consent is not considered a wrong and asking for consent can thus serve no enabling function.^24^ Where it *is* required, then we have identified three relatively distinct points on a graduated spectrum towards more demanding signalling standards. While the signalling standards are fixed points on that spectrum, the function served by consent will vary fully across it. As risks become more significant, and confidence declines that the use or disclosure enabled by consent is wanted, a more demanding signalling standard may be expected. The standard required in the context of either legal regime will depend on the distribution of emphasis between safeguarding and enabling functions across different legal principles (including, but not limited to, consent) and the significance of these functions in the context of other considerations. To put this another way, the rigour of the signalling standard will likely be shaped by safeguarding and enabling need, whether that need is otherwise met, and whether it is moderated by other considerations in the context.

We turn now to consider legal requirements and professional guidance regarding a valid consent to use and disclose patient information in the context of health care (as well, to a lesser extent, the context of health research). Locating each against our framework, as part of an investigation into conceptual alignment, helps to highlight inconsistency and ambiguity within and between professional guidance on the CLDoC, and between the CLDoC and data protection law. It also offers a perspective on the function that consent may serve in each case. Understanding this will prove important to an assessment of how to coordinate these regimes and bring them into more synchronous parallel, that is, better normative alignment. Before further exploring this, we need to be confident that we are comparing like with like: is the material scope of the CLDoC and data protection law the same? We begin by comparing the concept of ‘confidential patient information’ with ‘personal (health) data’.

## III. CONSENT—LEGAL REQUIREMENTS AND PROFESSIONAL GUIDANCE

### A. ‘Confidential patient information’ versus ‘personal (health) data’

The meaning of confidential information has not received much helpful definition in the common law. It has been defined rather circularly as any information that has ‘the necessary quality of confidence about it’.^25^ When discussing a context where the information was of a personal (as opposed to eg commercial) nature, in *Campbell v MGN*, Lord Hope opined:

The underlying question in all cases where it is alleged that there has been a breach of the duty of confidence is whether the information that was disclosed was private and not public. There must be some interest of a private nature that the claimant wishes to protect.^26^

The requirement that information be ‘private and not public’ may be understood to distinguish the category of confidential patient information from the category of ‘personal data’ in material ways.

Personal data is defined by the GDPR as constituting ‘*any* information relating to an identified or identifiable natural person (“data subject”)’.^27^ ‘Data concerning health’ (or health data, to use better shorthand) is defined in the GDPR as ‘personal data related to the physical or mental health of a natural person, including the provision of health care services, which reveal information about his or her health status’.^28^ Personal health data falls within the class of special category personal data. We discuss later the additional protections that apply to special category personal data.

The statutory definition of personal data appears distinguished from the CLDoC concept of confidential information across two axes: (i) it is broader across one axis as ‘personal’ may include *any* information relating to an identifiable^29^ person and can include information that is public, such as a person’s name, but (ii) it is narrower across the other as personal data must relate to a natural (living) person. Conversely, a duty of confidentiality may extend after an individual’s death.^30^ Moreover, data protection law applies only to the ‘processing of personal data wholly or partly by automated means and to the processing other than by automated means of personal data which form part of a filing system or are intended to form part of a filing system’.^31^ It does not, in other words, cover personal data that is divulged only in oral form; the CLDoC, conversely, covers all communication forms, including information that is relayed only through oral form.

Despite these differences, there are reasons to doubt that the boundaries mapped by definitions of personal (health) data and judicial description of ‘confidential information’ are as radically different as might first appear.^32^ Even if the outer boundaries do extend in different directions, we take for the purposes of our argument an example of data that would sit clearly in the nexus. We are concerned with health data relating to an identified individual (patient), gathered during the course of providing care, and held in written form, eg in a health record.^33^ The courts have previously found that such health data is ‘obviously private’ and protected under the CLDoC.^34^ This also clearly falls within data protection law’s definition of personal data and, indeed, within the narrower class of special category personal data. Thus, while the legal terms ‘confidential information’ and ‘personal (health) data’ may not overlap perfectly,^35^ for the purpose of this article’s focus on patient information, it can be stated that personal health data gathered and used by a health professional providing care to a patient will fall within both regimes.

We now turn to consider the requirements of a valid signal of consent in each regime. We do not seek to consider all the questions raised by a valid consent. The literature on consent is already voluminous and some issues, including the issues associated with appropriately informing a consent under the CLDoC and the communication of a child’s consent under data protection law, we have ourselves considered elsewhere.^36^ Here, we focus on an issue that has hitherto been relatively neglected: the (non)alignment between the signalling standards of a valid consent under the CLDoC and data protection law to uses of patient information in a health care (and health research) context. While specific, it is important given the significance of the data routinely captured and processed within the health care system in the UK. It also provides an important illustration of what may be required for the CLDoC and data protection law to work together more generally.

### B. Requirements of Valid Consent under each of the Regimes

As already noted, it is relatively uncontroversial to assert that for consent to be valid, it must be appropriately informed, freely given, and communicated by someone with capacity. However, the threshold requirements attached to these elements can vary between areas of law, and interpretation of requirements in different circumstances can be controversial.^37^ We focus in this section on the distinction that can be drawn between how a valid consent may be *signalled* under, respectively, the CLDoC and data protection law, with a view to determining the extent to which the requirements are conceptually aligned—and the implications for interplay between the regimes.

#### 1. Consent to disclose confidential patient information under the CLDoC

In *Hunter v Mann*, Boreham J opined that ‘ … in common with other professional men … the doctor is under a duty not to disclose [voluntarily], without the consent of his patient, information which he, the doctor, has gained in his professional capacity, save … in very exceptional circumstances’.^38^ While consent is not the only way in which a breach of confidentiality may be avoided, and indeed there are other grounds for a justified disclosure, as professional guidelines indicate, it is a routinely relied upon justification to use and disclose confidential patient information for the purposes of patient care or health research.

Of the five grounds that the current General Medical Council (GMC) *Confidentiality* guidelines^39^ explain are available, the first two are grounded in patient consent:

Confidentiality is an important ethical and legal duty but it is not absolute. You may disclose personal information without breaching duties of confidentiality when any of the following circumstances applies.

- The patient *consents*, *whether implicitly or explicitly*, for the sake of their *own care or for local clinical audit* […].
- The patient has given their *explicit consent* to disclosure for *other purposes* […].^40^

The guidelines thus draw a distinction between purposes (with care and local audit distinguished from other purposes, including health research) and link them to different types of signal standard. While the tripartite elements of a valid consent must still be present—appropriately informed, freely given, and communicated by someone with capacity—the *mode* of signalling required varies depending upon the *purpose* (though the safeguarding and enabling functions may be seen throughout). We now proceed to unpack these standards according to purpose.

##### a. Purpose of direct care or local clinical audit: Implied Consent

Implied consent is not a new concept in medical law; it can be traced back at least as far as the classic 19th century case of *O’Brien v Cunard SS Co*, where the Supreme Judicial Court of Massachusetts held that when consent is used as a defence to an assault action, the totality of the circumstances must be considered. In this particular case, rolling up one’s sleeve to receive a vaccination and not objecting at any point to the vaccination clearly indicated an overt act and an implied consent to receive the vaccination.^41^ This kind of act would most clearly align with what we have described as Level 2 (active implied), where for a patient’s information to be lawfully used and disclosed, the patient must signal consent through an unambiguous action. As we have already seen, however, consent may be implied by conduct in different ways short of statement. We have distinguished between Level 1 (passive implied), where conduct may be ambiguous to data use or disclosure specifically but is implied by general conduct (and no objection) in the context and Level 2, where agreement to data use or disclosure is unambiguously signalled by an overt act. Unfortunately, professional guidance does not clearly indicate which (Level) is sufficient for consent to be implied under the CLDoC, nor does such guidance recognise that the conceptual fit (with either Level) might vary between use and disclosure for purposes of direct care and local clinical audit (as the functions of safeguarding and enabling and mix with other considerations may shift).^42^

The 2013 independent report led by Dame Fiona Caldicott, *Information: To Share or not to Share? The Information Governance Review*, concluded in part:

To allow safe and effective inter professional and organisational sharing it is imperative that regulators agree a consistent language to describe the common set of conditions when implied consent can be relied upon by all parts of the health and social care system, including professionals, commissioners and providers.^43^

Still, to date, professional guidance does not make clear whether a Level 1 or Level 2 implied consent is sufficient to that ‘common set of conditions’.^44^

The GMC *Confidentiality* guidelines, quoted above, state that patient consent, *whether implicit or explicit*, is sufficient to justify disclosure for the purposes of a patient’s own care or for local clinical audit. According to the guidelines, doctors may rely on an implied consent to access relevant information about the patient or to share it with those who provide (or support the provision of) direct care to the patient if all of the following criteria are met:

1. the doctor is accessing the information to provide or support the individual patient’s direct care, or is satisfied that the person they are sharing the information with is accessing or receiving it for this purpose;
2. information is readily available to patients, explaining how their information will be used and that they have the right to object. (This can be provided in leaflets and posters, on websites, and face to face. It should be tailored to patients’ identified communication requirements as far as practicable.);
3. the doctor has no reason to believe the patient has objected to the disclosure; and
4. the doctor is satisfied that anyone they disclose personal information to understands that they are giving it to them in confidence, which they must respect.^45^

Consent given implicitly is given in ‘circumstances in which it would be reasonable to infer that the patient agrees to the disclosure of their data to certain others, even though this has not been directly expressed’.^46^ This guidance, and the suggestion that consent need not be ‘directly expressed’, supports the view that Level 1 is sufficient. The GMC guidance does not suggest an overt act, such as a nod of a head, is required; much, though, turns on an interpretation of what it is ‘reasonable to infer’ in the circumstances. Is it reasonable to infer Level 1 signalling is sufficient only where risks are sufficiently low or otherwise effectively mitigated by other safeguards (eg by other professional or employment obligations)? Might it be sufficient (even if risks are non-trivial), if there is independent reason (beyond silence or inaction following notification and opportunity to object) to think this use or disclosure is wanted by the individual patient or in his or her best interests? Are other considerations, such as the public interest, relevant to the circumstances in which it would be reasonable to infer agreement? Our analysis has suggested that each may be relevant to the ‘common set of conditions’, but it would be useful for guidance to be clear on what may be taken into account when determining what it is ‘reasonable to infer’ in the circumstances.^47^ If the four criteria listed above are both necessary and sufficient, then it would seem Level 1 consent is enough.

In contrast with the GMC guidance, the NHS Code of Practice on Confidentiality position more overtly conflates Level 1 or Level 2. It defines implied consent as ‘patient agreement that has been *signalled* by behaviour of an informed patient’ (emphasis added).^48^ This suggests a Level 2 consent is required. However, the Code elsewhere states:

Where patients have been informed of: then explicit consent is not usually required for information disclosures needed to provide that healthcare. Even so, opportunities to check that patients understand what may happen and are content should be taken.

- the use and disclosure of their information associated with their healthcare; and
- the choices they have and the implications of choosing to limit how their information may be used or shared;

*Where the purpose is not directly concerned with the healthcare of a patient however, it would be wrong to assume consent*. Additional efforts to gain consent are required or alternative approaches that do not rely on identifiable information will need to be developed.^49^

So, is active signalling necessary? Guidance that it is *not* wrong to assume consent in circumstances where information about the use and disclosure has been given, and the patient has been informed of their choices, suggests not. At least, not ‘usually’. Opportunities to check patient understanding ‘should be taken’, but they need not *necessarily* have actively signalled consent through anything more than receipt of health care and inaction or silence in response to an opportunity to object to notified use and disclosure. Level 1 is sufficient.

It would be clearer, and more helpful, if professional guidance unambiguously indicated whether Level 1 was sufficient, or if an active signal (i.e. Level 2) is necessary. Guidance can, however, be reasonably interpreted to suggest that Level 1 may be sufficient for both care and local audit. Level 2 or Level 3 signal would be valid but would (‘usually’) surpass minimum requirements.

##### b. Other purposes (eg health research): Explicit Consent

GMC guidance to doctors advises they should ‘ask for explicit consent to disclose identifiable information about patients for purposes other than their care or local clinical audit, unless the disclosure is required by law or can be justified in the public interest’.^50^ Explicit consent is defined in the NHS Code of Practice on Confidentiality as meaning ‘articulated patient agreement’.^51^ The Code elaborates that explicit consent relates ‘to a clear and voluntary indication of preference or choice, usually given orally or in writing and freely given in circumstances where the available options and the consequences have been made clear’.^52^ The qualification that such consent is ‘*usually’* given orally or in writing does again introduce some uncertainty.^53^ Specifically, it suggests that Level 3 explicit consent is *usually* required, but occasionally Level 2 (active implied) consent might sometimes be sufficient.

Despite the uncertainty created by the qualifier ‘usually’, the use of the term ‘explicit’ most readily aligns with what we have described as a Level 3 consent, and is a more demanding signalling standard than professional guidance suggests is sufficient for direct care or local clinical audit. While recognising that the need for consent may be moderated or overridden by other considerations (in the form of legal requirement or public interest justification), this signalling standard indicates a view that, all other things being equal, there is a more significant safeguarding or enabling function to be played by consent in the context of disclosure for health research than direct care or local audit: authorising use or disclosure that would be a more significant wrong absent consent.

As we turn now to consider data protection law more closely, we see that, despite the relative clarity with regards to the signal standard of consent, consent is *not* recommended to be the normal means by which use or disclosure of patient information in the health care or health research context is justified.

#### 2. Consent to process personal (health) data under data protection law

Data protection law has long recognised consent to be one of a number of legal bases for processing personal data.^54^ Only since the implementation of the GDPR^55^ in 2018, though, has there been in-depth guidance from regulators (at least in the UK) on the implications of applying these various legal bases in the health care and health research contexts. The UK’s Health Research Authority, and more recently the European Data Protection Board,^56^ have advised against relying on consent for processing personal data when alternative legal bases exist because of concerns about the impact of withdrawal by data subjects on the research project, potential power imbalances between the data subject and controller, and concern that data subjects’ rights that follow from consent under the legislation cannot be applied because it would limit the validity of the research.^57^

There are two critical legal purposes that consent can play under data protection law. First, it can provide a legal basis to process personal data (under Article 6 GDPR). Data protection law requires that a legal basis be secured before any personal data may be processed. Six legal bases are possible, one of which is the consent of the data subject ‘for one or more specific purposes’.^58^ Second, it can establish an exception to the general prohibition on the processing of a special category of data, such as personal health data (under Article 9 of the GDPR).^59^ The requirements of a valid consent are stricter in relation to the second purpose than in relation to the first, presumably reflective of increased perceived risks for these special categories of personal data. We consider first the requirements of *any* consent under data protection law.

##### a. Consent to the processing of personal data

Consent is defined in the GDPR as ‘any freely given, specific, informed and unambiguous indication of the data subject’s wishes by which he or she, by a statement or by a clear affirmative action, signifies agreement to the processing of personal data relating to him or her’.^60^ Thus, the data protection law requirements for a valid consent see a slight expansion of the elements for validity. Such consent must be (1) freely given, (2) informed, (3) specific, and (4) unambiguously indicated by a statement or by a clear affirmative action (by someone with capacity).^61^ We take this last element of ‘indicated’ to be roughly synonymous with ‘signalled’. With respect to the condition of ‘specific’, the Article 29 Working Party^62^ has stated that this requires data controllers to apply: ‘(i) purpose specification as a safeguard against function creep, (ii) granularity in consent requests [i.e. specific consents for specific purposes], and (iii) clear separation of information related to obtaining consent for data processing activities from information about other matters’, ie controllers should provide specific information with each separate consent request about the data that are processed for each purpose, in order to make data subjects aware of the impact of the different choices they have.^63^

Recital 32 of the GDPR expounds upon the requirement for an unambiguous indication by statement or clear affirmative action:

Consent should be given by a *clear affirmative act* … such as by a written statement, including by electronic means, or an oral statement. This could include ticking a box when visiting an internet website … or another *statement or conduct which clearly indicates in this context the data subject’s acceptance of the proposed processing of his or her personal data*. Silence, pre-ticked boxes or inactivity should not therefore constitute consent.^64^

It is clear then that a Level 1 consent will not under any circumstances be a valid consent to process personal data. Article 6(1)(a) requires a clear affirmative act—an unambiguous signal—that could be achieved through conduct, eg the digital equivalent of rolling up one’s sleeve. This may be achieved by, for example, completing and returning a hard copy or electronic survey or questionnaire. In short, Level 2 consent is the minimum permissible level under data protection law.

##### b. Consent to the processing of personal health data

As noted, per Article 9 GDPR, data protection law applies particular requirements to the signalling of a consent to the processing of personal *health* data. Consent to the processing of special category data must be not only ‘unambiguous’ and indicated (ie signalled) by statement or clear affirmative action, it also must be ‘explicit’. The term ‘explicit’ is not defined in data protection law, but guidance issued by the UK’s Information Commissioner’s Office (ICO) states that ‘[t]he key difference is likely to be that “explicit” consent must be affirmed in a clear statement (whether oral or written)’. This corresponds to what we have described as Level 3 explicit consent.^65^

Guidance thus suggests that while consent to the processing of (regular) personal data may be expressed by statement *or* clear affirmative action, ie Level 2 active (implied) consent, consent to the processing of personal health data *must* be Level 3 explicit consent. However, the most significant point, we again stress, and which we proceed to unpack in the following section, given its implications for the possibility of both conceptual and normative alignment between the two legal regimes, is that *consent is not the normal justification* for data processing under data protection law in the context of health care and health research.^66^

### C. Processing without Consent under Data Protection Law

Regulatory and professional advice to data controllers is that, in the context of health care and health research, more suitable alternatives to consent may be available to serve as the legal basis for processing personal data (under Article 6 GDPR) and to provide an exception to the general prohibition on the processing of special category data, ie a condition under which such data can be lawfully processed (under Article 9 GDPR). We first analyse this in the context of processing data for direct care and local clinical audit, before turning to the context of health research.

#### 1. Direct care and local clinical audit

When the GDPR was coming into force in mid-2018, guidance was issued to the UK health and social care system by the Information Governance Alliance (IGA).^67^ A key message was that: ‘Consent is one way to comply with the GDPR, but it is not the only way, and in many health and social care contexts obtaining GDPR-compliant consent (which is stricter than that required for confidentiality) may not be possible.’^68^

The guidance continues: ‘Organisations should consider the other conditions available before choosing to rely on consent.’^69^ It then goes on to ‘highlight the alternatives’.^70^ Under Article 6, other suitable bases might be Article 6(1)(d)—where processing is necessary in order to protect the vital interests of the data subject (eg the emergency life-or-death treatment scenario)—or Article 6(1)(e)—processing is necessary for the performance of a task carried out in the public interest or in the exercise of official authority vested in the controller (eg data processing carried out by a government body as part of its remit), which would include the scenario of routine health care provided within the NHS.^71^

Under Article 9(2), other suitable exceptions to process health data might be for the provision of health or social care,^72^ where processing is necessary to protect the vital interests of the data subject or of another natural person where the data subject is physically or legally incapable of giving consent,^73^ processing is necessary for reasons of substantial public interest on the basis of UK law,^74^ or processing is necessary for reasons of public interest in the area of public health, on the basis of UK law.^75^

Again, this ground would cover most instances of routine health care provided within the NHS. Further alternatives for processing health data in the health context also include Article 9(2)(c)—processing is necessary to protect the vital interests of the data subject or of another natural person where the data subject is physically or legally incapable of giving consent; Article 9(2)(g)—processing is necessary for reasons of substantial public interest, on the basis of domestic law; and Article 9(2)(i)—processing is necessary for reasons of public interest in the area of public health, on the basis of UK law.

There is still an obligation to provide information about processing and to respect a data subject’s right to object. Thus, even though consent is not relied upon to justify processing, there is, under our framework, a Level 1 (‘opt out’) consent operating in the background. It is not relied upon to justify the processing and not recognised as a consent in this context. Drawing attention to it can, however, help to understand how the regimes might achieve normative synchronicity, and discharge the functions of ‘safeguarding’ or ‘enabling’ in equivalent fashion, despite different signalling standards.

#### 2. Health research

IGA guidance refers the reader to Health Research Authority (HRA) guidance on the GDPR.^76^ The HRA advises that: ‘For the purposes of the GDPR, the legal basis for processing data for health and social care research should NOT be consent. This means that requirements in the GDPR relating to consent do NOT apply to health and care research.’^77^ The unstated rationale is that a research participant’s data protection rights (the safeguarding function) and autonomy interests (the enabling function) will be promoted (or at least respected) through other legal bases under the GDPR and their respective requirements.

Where processing is carried out by a public authority, such as a hospital or university, it will typically be possible to rely upon Article 6(1)(e) to satisfy the requirement for a legal basis: processing is necessary for the performance of a task carried out in the public interest or in the exercise of official authority vested in the controller.

The requirement for an exception to the default prohibition on the processing of special categories of data can be met, according to Article 9(2)(j), where:

processing is necessary for archiving purposes in the public interest, scientific or historical research purposes or statistical purposes in accordance with Article 89(1) (as supplemented by section 19 of the 2018 Act) based on domestic law, and which must be proportionate to the aim pursued, respect the essence of the right to data protection and provide for suitable and specific measures to safeguard the fundamental rights and the interests of the data subject.

As indicated, the UK’s Data Protection Act 2018 expressly permits special categories of personal data, such as health data, to be processed for scientific research purposes provided that the research is in the public interest and is in accordance with Article 89(1) of the GDPR as supplemented by section 19 of the Act.^78^ Such processing is not allowed if it is likely to cause substantial damage or substantial distress to a data subject; where the processing is carried out for the purposes of measures or decisions with respect to a *particular* data subject, it must be as part of a medical research project approved by an NHS research ethics committee.^79^ Again, then, while consent is not centre stage, there are other substantive safeguards operating to protect data subjects from unjustified risk and to enable uses of data that are deemed necessary and proportionate to their interests.

### D. What are the Implications of the Different Requirements of Consent under the CLDoC and Data Protection Law?

To summarise the position described so far: consent is the normal justification for use or disclosure of patient information in the context of health care and health research under the CLDoC, but *not* under data protection law. Professional guidance states that implicit or explicit consent may be relied upon to satisfy CLDoC obligations if using or disclosing confidential patient information for the purposes of direct care or local audit. There is mixed messaging, however, with regards to the need for a valid implied consent to be signalled. A reasonable interpretation of guidance is that Level 1 is sufficient. Guidance is clear that Level 3 consent is required for secondary purposes, such as health research.

The signalling standard of a valid consent in law and professional guidance is clearer in data protection law than in the CLDoC and, as it would appear, is not conceptually aligned with the CLDoC. Data protection law does not use the language of ‘implied consent’, but it does distinguish between ‘consent’ and ‘explicit consent’. At least a Level 2 consent is required to provide a legal basis for processing any personal data (under Article 6, unless an alternative to consent is available). If processing *health* data, then Level 3 consent is required (under Article 9, unless an alternative is available). This requirement applies whether health data is processed for the purposes of direct care, local clinical audit, or health research. This results in a misalignment between the signalling standards under the CLDoC and data protection law.

While signalling standards under data protection law are more rigorous than under the CLDoC, consent is, as we have stressed, less relevant to data protection compliance in the context of health care and research. This avoids the risk that a rigorous consent requirement in data protection law impedes crucial flows of data; the safeguarding and enabling functions are instead performed by checks and balances built into alternative legal bases to use personal health data. This includes what is equivalent to a Level 1 consent operating in the background. It is a relevant consideration under both regimes whether the processing of data is ‘fair’ and consistent with an individual’s reasonable expectations in the circumstances.^80^

The fact that signalling standards are clearest in relation to GDPR consent, which is *not* the normal justification, and least clear in relation to CLDoC consent, which *is* the normal justification, together with the fact that standards are not conceptually aligned across legal regimes operating in the same space, gives rise to the potential for confusion and complexity expressed by the NDG (and in turn, health professionals) over the past few years. The attempts to address this through professional guidance have not provided much clarity.

The GMC *Confidentiality* guidelines state, for example:

The standard of consent under the GDPR is higher than under the common law of confidentiality.

…

It will not always be appropriate for data controllers to rely on consent under GDPR as a condition for processing health data. For example, implied consent is an accepted concept under the law of confidentiality, but it is unlikely to be a sufficient basis for sharing personal data based on consent under Article 6(1)(a) of the GDPR, and will not be sufficient for sharing ‘special category data’ based on explicit consent under Article 9(2)(a) of the GDPR. However, the GDPR does provide alternative conditions for processing data which are likely to be more appropriate in a health context.

This means that a doctor who is a data controller may be relying on different legal justifications for disclosing information under the common law duty of confidence and under the GDPR. It also means that doctors can continue to share information on the basis of implied consent if the conditions set out in paragraphs 28 and 29 (for direct care) and 96 (for local clinical audit) of this guidance are met.^81^

What this suggests is that health professionals (foremost, clinicians) must be mindful that two legal regimes (not to mention possibly a third regime, being tort law) govern the lawful use of confidential patient information, and that while they may rely upon consent for one regime (CLDoC), they might not, if not *should* not, for another. But how are health professionals to safely navigate this divergent approach across the two legal regimes in the context of the purposes of direct care and local clinical audit or disclosure for health research? Are there any circumstances in which health professionals can rely on a valid consent to share confidential information across both regimes, and if so, what would such a consent look like? Is there no interplay between the regimes so that a consent obtained in relation to one is entirely inert in relation to the other? Do they operate entirely independently in normatively asynchronous parallel? We now turn to address such questions, focusing first on the context of direct care or local clinical audit, before turning to the health research context.

#### 1. Implied consent for direct care or local clinical audit

In this section, we provide clarity on the implications of the different requirements of consent under the CLDoC and data protection law, and what it means in practice in the context of using confidential patient information (personal health data) for the purpose of direct care or local clinical audit. As we have stated above, a Level 1 consent will *not* meet the requirements for consent under the GDPR, be it Article 6(1)(a) or Article 9(2)(a). A Level 2 consent would, however, in our view (and as we now proceed to argue), meet the requirements for consent under Article 6(1)(a) to process *non*-special category data (contrary to the view expressed in the GMC *Confidentiality* guidelines).

There is an enduring value to consent under the CLDoC even when processing data on a legal basis (and exemption) other than consent for the purposes of data protection law. If consent for direct care or local clinical audit is implied by conduct (be it Level 1 or Level 2), then one way to navigate the divergent conceptual approaches is simply to be clear that different standards apply under each regime and, as the GMC *Confidentiality* guidelines suggest, rely upon them accordingly. If proceeding thus, it would be easy to conceive of them operating entirely independently: with consent obtained under one regime being entirely irrelevant with regards to one’s responsibilities under the other. We suggest that this approach would be a missed opportunity and fail to recognise not only how the regimes seek to protect similar interests and values—even with divergent signalling standards—but also, importantly, the possibilities of legally significant interaction.

Without seeking to perfectly align signalling standards, it may nevertheless be possible to achieve *some* semblance of normative synchronicity and to better articulate how the two regimes may work together rather than in asynchronous parallel. While the approach taken to proscribing unfair or unreasonable uses of personal information is radically different between the two regimes, this does not mean they land in different places when trading off private and public interests and performing the functions of ‘safeguarding’ and ‘enabling’ in context. What varies between them is how consent is situated within distinct legal traditions and resultant matrices of safeguards and enabling capacities.^82^

The common law traditionally only intervenes in cases of misuse. Under the CLDoC, in determining what constitutes a misuse of personal information, the courts will take into account all circumstances of the case. Consent, its absence, or whether it can be inferred, is taken into account—but it is just one part of a bigger picture. Whether the use or disclosure of personal information constitutes a misuse in a given circumstance has been traditionally determined by an application of principle that affords judicial discretion to proscribe inequitable, or unconscionable, behaviour—now perhaps better described as behaviour that constitutes (wholly or severally) a (tort of) misuse of private information or which interferes with a reasonable expectation of privacy.^83^ The inherent flexibility is, in part, likely reason for the lack of clarity in professional guidance. Much will depend on what is perceived to be fair and reasonable in the circumstances and this will take into account the extent to which the use of data in the health care context aligns with, and is indeed motivated by, an individual’s best interests.

Data protection law has been shaped by the Court of Justice of the European Union, among other courts (such as the European Court of Human Rights), which helps give data protection law practical traction, but the primary architecture in this regime is, we submit, rules crafted by legislators and codified in statute. For data protection law, alternative legal bases for processing are prospectively described and detailed. Consent, rather than a relevant feature of circumstances, is an *alternative* legal basis. With a valid consent under data protection law, it is possible for an individual to accept risks that would not otherwise be appropriate. This may go some way to explain why the signalling standard is so rigorous *and* why consent is not considered the appropriate legal basis when more suitable alternatives exist. Consent is a legal basis that appears, at times, to tilt towards the enabling rather than safeguarding function.^84^

To require a Level 3 consent under the CLDoC for direct care or local clinical audit, then, would undoubtedly obstruct appropriate use in the circumstances of direct care and local clinical audit; indeed, it may not be practicable to obtain an explicit consent in the circumstances. It is also not necessary from an enabling or safeguarding perspective given the broader regulatory environment: the risks are effectively mitigated in the health care context (foremost through professional obligations and NHS operating standards) in ways that may make it riskier from the patient’s perspective *not* to use the data.

So, in summary, a Level 2 (implied) consent under the CLDoC *could* satisfy the requirements for consent as the legal basis of processing under Article 6(1)(a) GDPR and thus serve as the legal basis for processing under data protection law *and* authorise use under the CLDoC. The regimes would be bridged—in part. This might help illuminate the significance of a withdrawal of patient consent (as we discuss further below in Section IV), but would otherwise, we admit, have little practical effect. A Level 1 consent under the CLDoC is indicated as being sufficient in professional guidance and would not meet the threshold of a valid ‘consent’ under data protection law. Moreover, and more importantly, it is ‘health data’ that are being processed and the divide between (even Level 2) implied and (Level 3) explicit consent as the justification for processing personal health data, under Article 9(2)(a) of the GDPR, is too wide to be bridged.

Even if a Level 2 (implied) consent were to be obtained, it remains appropriate for a data controller to rely upon any of the ten conditions (ie exemptions) to lawfully process health data under Article 9(2), other than Level 3 explicit consent. As long as the exemption for processing is communicated clearly and transparently to a data subject, there is minimal chance that they will be misled about the function that consent under CLDoC is performing under data protection law. On the contrary, properly deployed, they may work effectively together if conceived in synchronous parallel to support *commonly conceived* reasonable expectations of ‘fair’ processing.

Here, we return to an important caveat that begins to recognise interplay between the two regimes. Any withdrawal of consent under the CLDoC—including an expressed objection—is not irrelevant under data protection law if it affects the lawfulness of processing.^85^  *If* a Level 2 consent has been relied upon to satisfy a CLDoC *and* to establish a legal basis for processing under data protection law, then the withdrawal of consent will clearly have an effect under both regimes. However, even if a Level 1 (or 2) implied consent has been relied upon to satisfy CLDoC requirements and something other than consent has been relied upon to establish a legal basis for processing under Article 6, such as Article 6(1)(d) or 6(1)(e), then withdrawal of consent may be legally significant from a data protection perspective. Indeed, it is a requirement of data protection law that all data processing be ‘lawful’.^86^

Here, we see a benefit of achieving normative synchronicity under both regimes. If they operate in synchronous parallel, then it will be unlawful to process data under data protection law in normatively similar circumstances to that under which it would be unlawful to (continue to) use patient information under the CLDoC. Continued use of patient information—despite withdrawal of consent—*can* be justified under the common law.^87^ Substantive normative synchronicity could be achieved if continued use were legally permissible under the CLDoC in circumstances where data protection law would also permit processing despite a withdrawal of consent or data subject objection to processing.^88^

We noted above that, even where consent is not relied upon to justify processing, there is something akin to a Level 1 consent operating in the background in data protection law, through the data subject rights of notification^89^ and objection. Regarding the latter, Article 21 GDPR establishes that a data subject shall

Have the right to object, on grounds relating to his or her personal situation, at any time to processing of personal data concerning him or her which is based on point (e) or (f) of Article 6(1).

This is not an absolute right, and there are circumstances where a data controller need not cease processing in the face of an objection. These include where ‘the controller demonstrates compelling legitimate grounds for the processing which override the interests, rights and freedoms of the data subject or for the establishment, exercise or defence of legal claims’.^90^

If operating in normative synchronicity, then an objection (under data protection law) is only overridden where this would be consistent with a reasonable expectation of privacy in the circumstances (under the CLDoC).^91^ Interpreting and applying the requirements of the two regimes in this way would allow them to operate in a way that was mutually supportive: protecting a common understanding of what constitutes ‘fair processing’ and reasonable expectations regarding use and disclosure of patient information. It would not require conceptual (re)alignment of existing signalling standards, but it would require an effort to progress the law in a concerted fashion rather than simply regarding the two regimes to operate in asynchronous parallel. There is already interplay as data protection law requires processing to be lawful. There are advantages to that interplay being clearly understood and normatively coherent.

#### 2. Explicit consent for research purposes

In this section, we proceed to provide clarity on the implications of the different requirements of consent under the CLDoC and data protection law, and what it means in practice in the context of using confidential patient information (personal health data) for the purpose of health research. Where a data controller is reliant on a legal basis and exemption other than consent for the purposes of satisfying requirements under Article 6 and Article 9 GDPR, respectively, but an explicit consent is relied on to lawfully disclose for health research under the CLDoC, there remains a responsibility under both regimes (already noted above) to use and disclose data fairly and in a way that is consistent with an individual’s reasonable expectations in the circumstances. Here, too, there is an opportunity to achieve normative synchronicity in a way that means interactions between them are normatively frictionless.

As in the case of direct care, this might be illustrated by considering the implications under data protection law for a withdrawal of explicit consent (under the CLDoC) to disclosure for research purposes. Once again, we argue such withdrawal might not trigger obligations under Article 21 GDPR but ought, at least, to be resolved consistently with Article 21. We reiterate that under neither regime is a withdrawal of consent, nor an expressed objection, necessarily determinative. It is possible that there may be a public interest justification available under the CLDoC, irrespective of any withdrawal of consent. Similarly, a data controller may justify continued processing for a research purpose ‘where necessary for the performance of a task carried out in the public interest’.^92^ The advantage of recognising how they may operate together, rather than simply alongside one another, is that an act with normative implications under one regime can be meaningfully mapped across to the other. This may facilitate productive interaction rather than normative inconsistency.

Having now explored the interaction between the two regimes and the implications without conceptual alignment of signalling standards, in the final Section IV, we turn to consider whether there would be any advantage to moving from the current situation to one where the requirements of a valid consent are more fully aligned.

## IV. BRIDGING THE DIVIDE—MOVING TO FULLY ALIGNED STANDARDS?

That professional guidance urges legal bases other than consent for health data processing, and that consent signalling requirements differ under the CLDoC and data protection law, risks confusion regarding the need for consent, and in turn, an understanding of how and where the ‘safeguarding’ and ‘enabling’ functions of consent are performed within the regulatory context. Demonstrating normative alignment helps, but the possibility of substantive (re)alignment of signalling standard across the conceptual axes should also be considered.

Prima facie, we think it preferable that the gap between the CLDoC and data protection law *be narrowed as far as reasonably possible* to develop a common standard across both regimes. Given the different roles played by consent under each regime, this possibility needs to be considered in the context of the role of consent more generally. We suggest that any move to align standards fully ought to be accompanied with an appreciation of the consequences of shifting what constitutes a normal justification for the use and disclosure of patient information. In the context of direct care and local clinical audit, that shift would be *away from* consent as the normal justification for use and disclosure. In the context of health research, it would need to be *toward* consent as the normal justification. In either case, such significant change ought only to be undertaken with a fuller consideration of implications beyond the signalling standard. In this section of our article, we can only begin that task. We hope, however, to indicate why it may be a worthy one to pursue.

As indicated above, there may already be scope for normative synchronicity. In both regimes, consent may be sought to protect and promote the safeguarding and enabling functions—so that health professionals and data controllers may exhibit respect for patients. However, consent is sought with an accompanying underlying message that other interests, including public interests, are in play. In neither regime does consent unilaterally determine either what is lawful or what is understood to be a ‘reasonable expectation’. Patients do not have absolute rights of control over their information in any area of law, and so long as good reasons can be demonstrated, it is possible that health professionals and data controllers can use patients’ information even without consent—and that this will always be done with the full protection of law.

Nevertheless, it would be possible to move to a position where there was not only normative synchronicity, but alignment across the signal standards themselves. Perhaps most obviously, the language of consent could be reserved for a situation where at least a Level 2 consent was in play. Moving the standards of consent under the CLDoC closer to those of data protection law, and in particular Article 6(1)(a), would provide greater reassurance that consent under the CLDoC is authentic: avoiding any reliance on consent where individuals are, in truth, unaware of the use and disclosure in question.^93^ This would be consistent with classic examples of implied consent—active expressions such as rolling up one’s sleeve or nodding one’s head. Also, it more clearly brings into play, where only Level 1 ‘consent’ is available, the need for other legal principles to perform a necessary safeguarding or enabling function, or to justify moderating or overriding the need for that function in the circumstances.

It does risk undermining appropriate use where Level 2 consent cannot be achieved (ie where Level 1 may currently be relied upon). Here, though, one of us has separately written about the possibility of foregrounding the test of ‘reasonable expectations’ in a way that continues to allow patient information to be used appropriately.^94^ We suggest that this may not require as much of a departure from the approach currently taken by the courts, when considering obligations under the CLDoC, as professional guidance would suggest. Indeed, we can already see something akin to this at work.

In the case of *R (W, X, Y and Z) v Secretary of State for Health and Secretary of State for the Home Department* [2015] EWCA Civ 1034,^95^ there was no argument that the patients had given consent (at any Level) to the disclosure of their information to the Home Office. The Court of Appeal did not frame analysis in this way, preferring instead to focus on whether there was a reasonable expectation of privacy in the relevant aspect in the circumstances. The Court considered it significant that it was good (read: sufficient) practice for individuals usually to be *notified* of the disclosure and therefore they were understood to have a choice as to whether to ‘accept the terms on which [treatment] was offered’.^96^ While not characterising such agreement as a (Level 1) consent, the Court did seem to find it significant that patients could be understood to have accepted the disclosure by not objecting at the time.^97^

In the circumstances, *without more*, a Level 1 signalling was not considered sufficient to establish consent as an explanation for the disclosure. We would agree with this position: there were non-trivial risks and yet no apparent genuine autonomous choice exercised by the patients. Other factors were taken into account by the Court beyond the two functions of safeguarding and enabling we have associated with consent. One of the merits of continuing to recognise that only a Level 1 ‘consent’ (under our framework) is operating is that it can help isolate and illustrate the need for further justification (as consent itself is not central to the justification) and allows alignment with data protection (and ethical) obligations regarding notification and objection.

While this would go some way to enabling alignment between the concepts of a valid consent under both the CLDoC and GDPR, it would not require an implied consent under the CLDoC to function as consent under data protection law. This is important to recognise as, while we have focused on the signalling standard, there are other reasons why it may be difficult to smoothly move from an implied consent under the CLDoC to consent (valid for Article 6) under the GDPR. Decentring consent as the normal justification under the CLDoC would, however, avoid these challenges.^98^

Our recommendation that the consent standard be thus aligned comes, therefore, with a significant qualification. Advantages to consistency, enabling more efficient communication, and alignment of signalling standard will only justify bridging the gap if this does not come at too great a cost with regards to uses of health data that are considered appropriate. Our suggestion is that, here, a robust standard of ‘reasonable expectation’ is developed as an alternative. To the extent this happens, we are arguing for consent to be decentred from the practice of compliance with the CLDoC as it appears to be decentred from the courts’ contemporary assessment of breach of the CLDoC.

While some might counter that the system has worked sufficiently well to now, we think the introduction of the GDPR and clearer (non-aligned) consent standards has shifted the legal landscape. Now, the risks of confusion between the two regimes operating in the same space—of crudely mapping the consent requirements of one to the other and losing much in translation—requires consideration of what could be done to have the requirements work constructively together. This is also a risk in the case of disclosure for health research purposes. Here, though, our recommendation is different. Rather than shifting from consent as the normal justification under the CLDoC, we suggest consideration is given to shifting *toward* consent as the normal justification for disclosure for the purposes of health research.

In our view, patients have a reasonable expectation that *if* they do provide *explicit* consent to the disclosure of confidential patient information, then that should also be the legal basis and condition upon which their personal (health) data is processed—unless they are explicitly informed otherwise at the time of providing that consent that another legal basis and condition is to be used, and sufficient justification is provided for the alternative. In other words, the rebuttable presumption should be that data controllers operate on the basis of explicit consent if explicit consent has been sought and provided. Indeed, it is potentially misleading and disingenuous to seek consent from patients to use their data only to inform them at a later stage—for apparently technical arcane reasons—that consent was not, in fact, performing the safeguarding and enabling function that they might reasonably expect.

Patients should not be expected to grasp, let alone entertain, the legal nuances of two disparate legal regimes. Only if there are compelling reasons to forego explicit consent, demonstrating both safeguarding and enabling functions to either be overridden or otherwise achieved, should a different legal basis be used under data protection law. We argue for this position because the conditions under which explicit consent operates are substantially equivalent under both regimes. In both regimes, patients/data subjects must be informed of the reasons why their otherwise confidential information is to be disclosed and must also be informed of other crucial information to make the consent an informed one, which is then signalled by an express statement.^99^ The alternative will create unnecessary confusion amongst stakeholders and may undermine the functions of consent under the CLDoC.

The consequences of relying on consent are not problem-free, particularly when thinking about the scenario whereby a patient withdraws consent to the disclosure of their information. It is to be remembered that if explicit consent is relied upon to justify disclosure under the CLDoC, and that is withdrawn, then continued disclosure may already be unlawful from a data protection perspective. Again, what we call for is a more express normative alignment to enable more coherent interaction where interplay already exists.

If explicit consent were relied upon under data protection law, and a patient/data subject were to withdraw that consent to the (ongoing) use or disclosure, there may be alternative grounds on which the health care professional/data controller can rely to continue disclosing or processing the information—provided that it falls within the reasonable expectations of the patient.^100^ This would be in the health research context, for example, where GDPR Articles 9(2)(j) and 89(1) could apply, and in the care context where Article 9(2)(h) could apply. Shifting the legal basis from consent to another to process personal (health) data must be fair, and the circumstances in which this may occur are narrow. As the ICO has noted in guidance to data controllers:

If you find at a later date that your chosen basis was actually inappropriate, it will be difficult to simply swap to a different one. Even if a different basis could have applied from the start, retrospectively switching lawful basis is likely to be inherently unfair to the individual and lead to breaches of accountability and transparency requirements.

It is therefore important to thoroughly assess upfront which basis is appropriate and document this. It may be possible that more than one basis applies to the processing because you have more than one purpose, and if this is the case then you should make this clear from the start.

If there is a genuine change in circumstances or you have a new and unanticipated purpose which means there is a good reason to review your lawful basis and make a change, you need to inform the individual and document the change.^101^

A ‘genuine change in circumstances’ or ‘a new and unanticipated purpose’ may both be applicable in the health context where a patient withdraws their consent, but processing of their health data is important either for their own health or for improving the health care system. Provided this shift in legal basis under Article 6(1) and condition under Article 9(2) is clearly communicated to the patient (or research participant)—and ideally part of the initial information-giving to obtain consent in the first place (explaining that things might change over the course of the data processing activity)—and provided that the previous legal basis of explicit consent under both the CLDoC and data protection law no longer applies, either because the consent has been withdrawn or because the purposes for processing the personal (health) data have changed and require a new legal basis, this should be permitted where fair^102^ and consistent with reasonable expectations^103^ and indeed, more arguably, *promoted*, to maximise value of patient data.^104^

## V. CONCLUSION

In this article, we explored the lack of clarity regarding the interplay of the CLDoC and the statutory law of data protection from the perspective of a health care professional engaged in clinical practice and research in the UK. Such professionals are under a responsibility to respect a duty of confidentiality owed to their patients and are employed by organisations with responsibilities under data protection law. We considered the possibility of bridging the divide between the CLDoC and data protection law by focusing on consent as the basis for use or disclosure of patient information, in particular, drawing together the relevant signals of a valid consent.

We have found that, while signalling standards are currently largely unaligned, there are opportunities for normative consistency. This would promote coherent interaction. It depends on improved clarity regarding existing standards, how differences are mitigated normatively within respective contexts, and how the regimes might thus work together rather than in asynchronous parallel. This is dependent on improved professional guidance on (non)alignment and interaction between the two regimes. Moving towards alignment of signalling standards across both normative and conceptual axes is possible, but only recommended with caveats, including a shift to, or from, consent as the normal justification for use or disclosure.

More specifically:

1. A lack of clarity in professional guidance is revealed if one distinguishes between Level 1 and Level 2 consent. However, it is clear that there is a stark contrast between the signalling requirements of a Level 3 (explicit) consent under data protection law (necessary to justify processing health data on the grounds of consent) and Level 1 or Level 2 (implied) consent. A reasonable interpretation of guidance is that a Level 1 signal is sufficient for the use and disclosure of patient information for direct care and local clinical audit under the CLDoC, with Level 2 or 3 valid but ‘usually’ unnecessary.
2. Under the status quo, with relatively unaligned standards, it is possible theoretically to align Level 2 active implied consent under the CLDoC and consent under data protection law for processing (non-special category) personal data under Article 6 GDPR. There may be some advantages to acknowledging such partial bridging from the perspective of conceptual clarity, even if it would make little practical difference in the medium term: consent, explicit or otherwise, is not normally relied upon for data protection purposes in the health context and Level 1 consent is considered by some professional guidelines as sufficient (and advisable) to justify use and disclosure of patient information for direct care and local clinical audit under the CLDoC. If Level 2 consent, sufficient for an Article 6(1)(a) consent, is de facto obtained, then it strengthens any argument that a withdrawal of consent should have effect under both regimes. We have, however, argued that a withdrawal of consent (even if Level 1) should act as equivalent to an objection to data processing under data protection law if they are to be normatively aligned.
3. Conceptual clarity would be further improved (and partial bridging secured) if Level 1 passive implied consent were no longer recognised in professional guidelines to be necessary or appropriate under the CLDoC. Disclosure of confidential patient information that currently takes place on the basis of Level 1 consent could alternatively be justified on grounds that disclosure did not interfere with a reasonable expectation of privacy. This would enable consistent guidance on consent requirements and bring to the fore consideration of other substantive controls serving a safeguarding and enabling function in the circumstances, such as a requirement for an independent reason to think that the use is necessary and proportionate to the individual’s interests. Maintaining normative synchronicity would, however, require a shift *away* from consent as the normal justification for use or disclosure under the CLDoC for the purposes of direct care or local clinical audit.
4. Where Level 1 or Level 2 consent is relied upon for disclosure (under the CLDoC), then it is not appropriate to seek an explicit consent to satisfy requirements of data protection law. However, where a Level 3 explicit consent is obtained for disclosure under the CLDoC for the purposes of health research, then such explicit consent also ought to be the default legal basis under data protection law for processing, both under Article 6(1) and as the relevant exemption under Article 9(2). It remains open for data controllers to rely on a legal basis and Article 9(2) exemption other than explicit consent, provided this is communicated transparently to the data subject before data processing takes place. Maintaining normative synchronicity would, however, require a shift *toward* consent being the normal justification for processing under data protection law for the purposes of health research.

Looking beyond the health context, the alignment challenges we focus on are not the only challenges: a similar set of challenges arises in respect of the interrelationship of ‘consent’ under contract law and the consent norms of data protection law. Perhaps, though, by charting limited alignment within the health context, we may in the future be able to uncover synergies between other contexts and legal regimes. Within the health context, even with limited alignment, we hope health professionals and regulators alike may be nudged to see closer links between the legal regimes with regard to the function of consent in the respective contexts and to recognise that this alignment, on the basis of consent, best protects and promotes fair processing and the reasonable expectations of patients. It remains the case, nevertheless, that in the absence of perfect and comprehensive alignment across the two regimes, we may find better avenues for bridging the two regimes through the use of legal bases other than consent, such as the public interest or disclosure that is in conformity with a patient’s reasonable expectation of privacy.

## Footnotes

1 Health and Social Care (National Data Guardian) Act 2018.

2 National Data Guardian, Twitter post (2 October 2019) at <https://twitter.com/NDGoffice/status/1179330436960575488> accessed 21 March 2021. It was not the first time that the NDG had made such an observation. See n 3 below.

3 The National Data Guardian (NDG) had noted as part of earlier consultation ‘some confusion about the need to respect both data protection and common law, *in particular with regard to the differing consent requirements of these areas of law*’ (emphasis added). See National Data Guardian, *National Data Guardian for Health and Social Care: a consultation about priorities* (18 February 2019), 6 at <https://www.gov.uk/government/consultations/national-data-guardian-a-consultation-on-priorities> accessed 21 March 2021. The response to the consultation included strong support for clarifying the interplay between the requirements of common law and data protection with the ‘most commonly requested piece of work … guidance on consent’. See National Data Guardian, *National Data Guardian for Health and Social Care: consultation response* (July 2019), 19–20 at <https://www.gov.uk/government/consultations/national-data-guardian-a-consultation-on-priorities> accessed 21 March 2021. See discussion in Section III below for elaboration on the ‘mixed messages’ currently received by those working in the field.

4 The CLDoC is sometimes referred to as the ‘duty of confidence’, and its breach a ‘breach of confidence’ (with the duty and cause of action originally arising from equity). See generally M Richardson and others, *Breach of Confidence: Social Origins and Modern Developments* (Edward Elgar, 2012). We use the term ‘Common Law Duty of Confidentiality’ in this article and treat it as synonymous with ‘duty of confidence’.

5 Some legal commentators distinguish implicit and implied consent. For example, Shaun Pattinson defines implicit consent as consent signalled through ‘suggestive conduct’, and implied consent as ‘a necessary means of fulfilling a purpose for which express consent has been obtained and the patient is reasonably expected to be aware of this connection’. See S Pattinson, *Medical Law and Ethics* (6th edn, Sweet & Maxwell 2020) 4-008. We agree there is an important conceptual difference between implied and implicit consent; our focus here is on the conduct of a patient and whether it may be interpreted in a way that suggests their consent is deemed valid. For the purpose of this article, we use the term ‘implied consent’ unless otherwise indicated.

6 Data Protection Act 2018, s 3(9), provides a definition of ‘data protection law’. The UK’s exit from the European Union has resulted in changes to Council Regulation (EU) 2016/679 of the European Parliament and of the Council of 27 April 2016 on the protection of natural persons with regard to the processing of personal data and on the free movement of such data, and repealing Directive 95/46/EC [2016] OJ L119/1 (hereinafter, the GDPR) and the Data Protection Act 2018. The revision of the GDPR, pursuant to the Data Protection, Privacy and Electronic Communications (Amendments etc) (EU Exit) Regulations 2019, as amended by the Data Protection, Privacy and Electronic Communications (Amendments etc)(EU Exit) Regulations 2020, means that the applied GDPR is now known as the ‘UK GDPR’. The analysis in this article, focusing on the signalling standards (on the data protection law side) in arts 6 and 9 GDPR, is not materially affected by the transition to a UK GDPR. More specifically, arts 6(1)(a) and 7 have not been amended, and art 9(2)(a) has been amended only to substitute the phrase ‘Union or Member State law provide’ with ‘domestic law provides’. Reference in this article to the ‘GDPR’, and unless otherwise indicated, may therefore be seen as interchangeably applicable to both the EU GDPR and UK GDPR. See generally, UK Information Commissioner’s Office (ICO), ‘UK GDPR’ at <https://ico.org.uk/for-organisations/dp-at-the-end-of-the-transition-period/data-protection-now-the-transition-period-has-ended/the-gdpr/> accessed 21 March 2021.

7 See, eg Health Research Authority, ‘Consent in Research’ (NHS Health Research Authority, Guidance, (19 April 2018) at <https://www.hra.nhs.uk/planning-and-improving-research/policies-standards-legislation/data-protection-and-information-governance/gdpr-guidance/what-law-says/consent-research/> accessed 21 March 2021).

8 There is guidance available on consent under each regime, eg guidance on consent and confidential patient information provided in the NHSX IG portal: NHSX, ‘Consent and confidential patient information’ (9 December 2020) at <https://www.nhsx.nhs.uk/information-governance/guidance/consent-and-confidential-patient-information/> accessed 21 March 2021, and the GMC guidance mentioned later (see n 30 below). While existing guidance may help professionals to understand how consent is understood under the different legal regimes, it does not disambiguate signal standards to the degree necessary to establish the extent of alignment or discuss the kind of interplay between them that we suggest necessary and seek to provide here.

9 See, eg P Carey, *Data Protection: A Practical Guide to UK Law* (6th edn, OUP 2020); M Taylor, ‘Confidentiality and Data Protection’ in J Laing and J McHale (eds), *Principles of Medical Law* (4th edn, OUP 2017). We suspect they have probably been asked, and answered, in practice by those having to grapple with health data governance in this context. It is not clear, however, whether they are consistently answered in the same way.

10 Direct care can be defined as ‘a clinical, social or public health activity concerned with the prevention, investigation and treatment of illness and the alleviation of suffering of individuals. It includes supporting individuals’ ability to function and improve their participation in life and society. It includes the assurance of safe and high quality care and treatment through local audit, the management of untoward or adverse incidents, person satisfaction including measurement of outcomes undertaken by one or more registered and regulated health or social care professionals and their team with whom the individual has a legitimate relationship for their care’. See Dame Fiona Caldicott, *Information: To Share or not to Share: The Information Governance Review* (National Health Service, March 2013), 128 at <https://www.gov.uk/government/publications/the-information-governance-review> accessed 21 March 2021.

11 For an argument in favour of consent as the normal justification under data protection law in the health research context, see E Dove and J Chen, ‘Should Consent for Data Processing be Privileged in Health Research? A Comparative Legal Analysis’ (2020) 10 International Data Privacy Law 117–31.

12 Our focus in this article is on the perspective of a health professional working in the health care system. We are not here discussing the data processing requirements as they apply to health research more generally, but rather, only as they apply to disclosure for research purposes by a health professional who has obtained data in the context of direct care.

13 J Berg and others, *Informed Consent: Legal Theory and Clinical Practice* (2nd edn, OUP 2001); D Beyleveld and R Brownsword, *Consent in the Law* (Hart Publishing 2007). As Beyleveld and Brownsword point out, the relevant ‘safeguard’ may work to protect the individual who receives consent (as a legal ‘flak-jacket’) as well as the individual who provides the consent. We are focused on the function of a safeguard to the individual who is asked for consent, where the requirement protects from an action to which they have not agreed.

14 Although, it is worth recognising two things. The first (as we again recognise later) is that *not* sharing data can also carry risks. For a useful overview of the harms that might follow from both using, and not using, health data, see G Laurie and others, ‘A Review of Evidence Relating to Harm Resulting from Uses of Health and Biomedical Data’ (Farr Institute; Mason Institute for Medicine, Life Sciences and the Law, 2014). See also Principle 7 of the Caldicott Principles: ‘The duty to share information for individual care is as important as the duty to protect patient confidentiality’, at <https://www.ukcgc.uk/manual/principles> accessed 21 March 2021. The second is that any assumption that personal health data carries with it a higher level of risk than other kinds of data is open to challenge. Such challenge may extend to the classification of different categories of data as necessarily ‘sensitive’. While we are sympathetic to such challenge, it remains the case that personal health information is accorded special status under both data protection law and the CLDoC. See also LA Bygrave, *Data Protection Law: Approaching Its Rationale, Logic and Limits* (Kluwer Law International 2002), especially ch 3 (discussing the controversy around the ‘sensitivity principle’).

15 Berg and others (n 13) above; Beyleveld and Brownsword (n 13) above.

16 T Beauchamp, ‘Autonomy and Consent’ in F Miller and A Wertheimer (eds), *The Ethics of Consent: Theory and Practice* (OUP 2010) 55–78; SAM McLean, *Autonomy, Consent and the Law* (Routledge 2010).

17 For discussion of public interest in the health research and data protection law context, see M Taylor and T Whitton, ‘Public Interest, Health Research and Data Protection Law: Establishing a Legitimate Trade-Off between Individual Control and Research Access to Health Data’ (2020) 9 Laws 6 (1–23).

18 For the sake of completeness, we acknowledge a fourth level: Level 0. At this level, no signal of consent is required as consent is not necessary. This is because (1) risks are *de minimis* (or otherwise mitigated to the point they are *de minimis*) or exposure to risk is not considered wrong in the circumstances, and (2) the relevant action can legally take place without consent. For example, my photograph is taken as I walk past a tourist attraction by a holiday maker snapping a street scene. I am not asked for consent to be included in the photograph, but photography would be considered appropriate in the circumstances because (1) if taking the photograph does expose me to a risk, then it is not considered to be a legal wrong, and (2) the photograph can lawfully be taken without my consent. There is no relevant safeguarding or enabling function performed by consent in the circumstances.

19 Though we shall say later that we consider such a term something of an oxymoron. Without a valid signal of consent, it can only be imputed in the circumstances and we question whether this is a genuine consent.

20 eg I walk into a private venue where it is clearly signed that promotional photography will take place. Here the conduct itself is ambiguous with regards consent to the photography specifically but, assuming equivalent alternative venues are available to me, I have an accessible means to signal my objection and my entrance thus reasonably implies at least acceptance. More burdensome signalling (eg requiring people to read and sign consent forms) is considered disproportionate and may itself be more autonomy intrusive than the act for which consent is sought.

21 eg I nod my head and smile for the camera when asked by the photographer if I am happy for my photograph to be taken and included in a story featuring in the local city blog, which is open to broad public consumption.

22 eg I sign a form expressly indicating my consent to my photograph being used in an advertising campaign for a controversial product.

23 See, eg National Health Service Act 2006, s 251; Health Service (Control of Patient Information) Regulations 2002, SI 2002/1438; Health and Social Care Act 2012; Health Protection (Notification) Regulations 2010, SI 2010/659; Reporting of Injuries, Diseases and Dangerous Occurrences Regulations 2013, SI 2013/1471. eg England's National Data Opt-Out, which was introduced on 25 May 2018, allows a patient to choose if they do not want their confidential patient information to be used for purposes beyond their individual care across the health and adult social care system in England, except where they have given their explicit consent to a specific use of their data (eg for a research project). As noted in the national data opt-out operational policy guidance document, the National Data Opt-Out ‘applies where section 251 support, which enables the use of confidential patient information without consent, is relied upon, and guidance is provided for health and care professionals in assessing what is [confidential patient information]’. Options for lawful disclosure under the common law include the use of the legal gateways set out in the Health Service (Control of Patient Information) Regulations 2002 (made under s 251 of the NHS Act 2006), which allow confidential patient information to be used without patient consent. See NHS Digital, ‘National Data Opt-Out Operational Policy Guidance Document. 2: What are National Data Opt-Outs?’ at <https://digital.nhs.uk/services/national-data-opt-out/operational-policy-guidance-document/what-are-national-data-opt-outs> accessed 21 March 2021.

24 At least, not legally. We must leave aside the question of the function of consent in enabling relationships more generally. It might be considered polite to ask, even if not legally required, and that etiquette might help to establish and consolidate a doctor–patient relationship. Here we are focused on the role of consent in the CLDoC and data protection law.

25 *Saltman Engineering Co Ltd v Campbell Engineering Co Ltd* (1948) 65 RPC 203, 215. In *Coco v A N Clark (Engineers) Ltd* [1968] FSR 415, Megarry J explained that such ‘information must be of a confidential nature’: at 419–20.

26 *Campbell v Mirror Group Newspapers Ltd* [2004] UKHL 22; [2004] 2 AC 457 at [92].

27 GDPR, art 4(1) (emphasis added). This definition is expanded to clarify that: ‘ … an identifiable natural person is one who can be identified, directly or indirectly, in particular by reference to an identifier such as a name, an identification number, location data, an online identifier or to one or more factors specific to the physical, physiological, genetic, mental, economic, cultural or social identity of that natural person’.

28 GDPR, art 4(15).

29 The statutory definition specifies that the person may be ‘identified’ as well as ‘identifiable’, but we understand that an identified person is de facto identifiable. NB: a person’s identifiability has also been found to be key to establishing the scope of a common law duty of confidentiality. See *R v Department of Health ex parte Source Informatics Ltd* [2000] 1 ALL ER 786. Although, for a critical view of this, see M Taylor, ‘*R v Department of Health, ex parte Source Informatics Ltd* [1999]’ in J Herring and J Wall (eds), *Landmark Cases in Medical Law* (Hart Publishing 2015).

30 See, eg General Medical Council, *Confidentiality: Good Practice in Handling Patient Information* (General Medical Council, 2018), [134] at <https://www.gmc-uk.org/ethical-guidance/ethical-guidance-for-doctors/confidentiality> accessed 21 March 2021. See also *Lewis v Secretary of State for Health* [2008] EWHC 2196; Freedom of Information (Scotland) Act 2002, s 38. We also note, however, that the GDPR permits national data protection laws to extend to personal data about deceased persons, as is the case, for example, in Denmark, which applies up to 10 years following the death of the deceased. See Denmark’s Act No 502 of 23 May 2018 (Act on supplementary provisions to the regulation on the protection of natural persons with regard to the processing of personal data and on the free movement of such data (the Data Protection Act), s 2(5) at <https://www.datatilsynet.dk/media/7753/danish-data-protection-act.pdf> accessed 21 March 2021. The UK GDPR does not apply to personal data about deceased persons.

31 GDPR, art 2(1).

32 The CLDoC has evolved since the Human Rights Act 1998 came into force. UK court rulings now indicate that in addition to the CLDoC, a tortious duty may also be imposed on all people (not just professionals) to not misuse private information, including information that may be in the public domain. See Lord Nicholl in *Campbell v MGN* (n 26) above, [14]. See also *Vidal-Hall v Google Inc* [2015] EWCA Civ 311; *Wood v Commissioner of Police for the Metropolis* [2009] EWCA Civ 414; *C v Chief Constable of the Police Service of Scotland* [2019] CSOH 48 (in which the Court of Session (Outer House) recognised a right of privacy in terms of the common law of Scotland). Thus, it may be said that there are in fact three distinct heads of law now at play: statutory data protection law, the CLDoC, and tort law. This remains an emerging area and as professional guidance for health professionals does not substantively cover the tort of misuse of private information as a part of a third legal regime to be mindful of, we leave this consideration aside for future discussion. However, we do note that this broadens an understanding of when an interest of a private nature may be engaged. The common law will protect private information not only in the context of a confidential relationship, but whenever a person has a reasonable expectation of privacy in all the circumstances. In the context of medical care, a reasonable expectation of privacy may be understood to extend to all personal data disclosed—including that which might be public knowledge in other circumstances. *R (W, X, Y and Z) v Secretary of State for Health and Secretary of State for the Home Department* [2015] EWCA Civ 1034. In the health care context at least, this narrows any gap between confidential information and ‘personal data’ under statutory data protection law. Closing the gap from the other direction, interpretation of the requirement that personal data *relate to* an individual may similarly bring the ontology of personal data and that of confidential or private information closer together. However, this approach has been criticised (see B Wong, ‘Delimiting the Concept of Personal Data after the GDPR’ (2019) 39 Legal Studies 517, 525) and is arguably at odds with the approach taken by the European Court of Justice (see *Peter Nowak v Data Protection Commissioner* (2017) Case C-434/16 (CJEU) [34]). The Court of Appeal in *Durant v Financial Services Authority* [2003] EWCA Civ 1746 [28] considered it useful when interpreting the material scope of the definition of personal data to consider whether ‘it is information that affects [Mr Durant’s] privacy’. See also *B v General Medical Council* [2018] EWCA Civ 1497 [37], [41]. Regardless of whether this continues to be the correct approach post-GDPR, it does illustrate how ‘personal’ has previously been interpreted as almost meaning the same as ‘private’ under data protection law: *JR60’s Application* [2013] NIQB 93 [29].

33 Here, we set aside for the purposes of this article’s scope, and as already indicated in *R (W, X, Y and Z)*, n 32 above, the important question of whether all or only some of the information within that health record constitutes either personal data or confidential patient information, such as demographic data and health indicators such as body mass index (BMI) or blood pressure.

34 *Campbell v MGN* (n 26) above.

35 Particularly in the Big Data era where the (health) significance of data comes from joining up multiple disparate sources.

36 See, eg, V Chico and M Taylor, ‘Using and Disclosing Confidential Patient Information and the English Common Law: What Are the Information Requirements of a Valid Consent?’ (2018) 26 Medical L Rev 51; M Taylor and others, ‘When Can the Child Speak for Herself? The Limits of Parental Consent in Data Protection Law for Health Research’ (2018) 26 Medical L Rev 369.

37 See, eg ibid.

38 *Hunter v Mann* [1974] 1 QB 767, 772.

39 General Medical Council (n 30) above.

40 ibid [9].

41 *O'Brien v Cunard SS Co*, 154 Mass. 272, 28 NE 266 (1891).

42 To take the case of local clinical audit as an example, the risks posed by such processing to a patient may be considered extremely low, perhaps in some cases *de minimis*, but they are not non-existent. Audit invariably involves disclosure of confidential patient information to a broader range of persons than would otherwise be necessary and some audits relate to conditions which may be particularly sensitive. One can reasonably doubt equivalence between a patient’s want for data to be used to provide care and use for audit purposes. Indeed, it is unlikely that everyone who receives medical treatment would consider themselves to have autonomously elected for their confidential patient information to be used for audit purposes. This does not mean it is not justified. As long as supplemented by other (public interest) considerations, and patients are safeguarded from more than negligible risk, a relatively trivial interference with autonomy may be dismissed as insignificant. This contrasts with the use of data for the purposes of direct care where a different mix of ‘enabling’, ‘safeguarding’, and other relevant considerations are likely to be in play due to alignment with patient’s wants and vital interests.

43 Fiona Caldicott (n 10) above, 38 (emphasis added).

44 And yet, from the perspective of the safeguarding and enabling functions, there is a significant difference between 1) a scenario in which a patient is putatively given notice (via posting of a privacy policy online or in hard copy at a surgery) and, absent objection, is taken to have (passively) implicitly consented to have their information used and shared with others for their care, and 2) a scenario in which a patient signals actively and unambiguously to the health professional, by a nod of the head or a thumbs up, that they permit to have their information used and shared with others.

45 General Medical Council (n 30) above, [28].

46 ibid [13].

47 And may feature in varying mixes for use and disclosure for direct care purposes and local clinical audit. The significance of this distinction may vary depending upon whether one is foregrounding the safeguarding or the enabling function of consent—and the respective level of perceived risk and fit with previously expressed preferences.

48 Department of Health, ‘Confidentiality: NHS Code of Practice’ (November 2003) at <https://www.gov.uk/government/publications/confidentiality-nhs-code-of-practice> accessed 21 March 2021 (NHS Code of Practice), 5.

49 ibid [15]–[16].

50 General Medical Council (n 30) above, [8(f)].

51 NHS Code of Practice (n 48) above, 5.

52 ibid.

53 It leaves ambiguous whether an actively signalled consent, short of verbalised statement, might constitute an explicit consent in some circumstances.

54 Data protection law requires that a legal basis be secured before any ‘personal data’ may be processed; personal data includes health information relating to a patient.

55 Under the GDPR, the requirements for consent have been tightened. The law provides a clearer definition, as compared to the previous Council Directive 95/46/EC of the European Parliament and of the Council of 24 October 1995 on the protection of individuals with regard to the processing of personal data and on the free movement of such data [1995] OJ L281/31 (1995 Data Protection Directive), of what consent constitutes and establishes enhanced conditions for a valid consent (art 7). Moreover, there is now a requirement for data controllers to specify the legal basis of processing in privacy notices to ‘data subjects’ (as reflected in GDPR, arts 12-14), be it consent or another legal basis.

56 European Data Protection Board, *EDPB Document on Response to the Request from the European Commission for Clarifications on the Consistent Application of the GDPR, Focusing on Health Research* (adopted 2 February 2021) paras 5–6.

57 See, eg Health Research Authority (n 7) above.

58 GDPR, art 6(1)(a).

59 We note that consent plays at least two other roles under the GDPR: (1) it permits data subjects to be subject to a decision based solely on automated processing, including profiling, which produces legal effects concerning them or similarly significantly affects them (art 22); and (2) it permits an international data transfer, assuming the data subject been informed of the possible risks of such transfers for the data subject due to the absence of an adequacy decision and appropriate safeguards (art 49). These additional roles, however, are not seen as critical for the purposes of this analysis.

60 GDPR, art 4(11).

61 See also European Data Protection Board, *Guidelines 05/2020 on consent under Regulation 2016/679, Version 1.0* (adopted 4 May 2020) 6.

62 An advisory body (now superseded by the European Data Protection Board) made up of a representative from the data protection authority of each EU Member State, the European Data Protection Supervisor, and the European Commission.

63 Art 29 Data Protection Working Party, *Guidelines on Consent under Regulation 2016/679* (adopted 10 April 2018) WP259 Rev.01 11-12.

64 GDPR, recital 32 (emphasis added). See also *Bundesverband der Verbraucherzentralen und Verbraucherverbände — Verbraucherzentrale Bundesverband eV v Planet49 GmbH* (2019) Case C-673/17 (CJEU); *Orange România SA* v *Autoritatea Naţională de Supraveghere a Prelucrării Datelor cu Caracter Personal (ANSPDCP)* (2020) Case C-61/19 (CJEU).

65 Information Commissioner’s Office, ‘What is Valid Consent?’ at <https://ico.org.uk/for-organisations/guide-to-data-protection/guide-to-the-general-data-protection-regulation-gdpr/consent/what-is-valid-consent/> accessed 21 March 2021.

66 While not recognised as conditions for valid consent under data protection law, the requirements for notification and the right to object to data processing, which are set out in data protection law as two distinct data subject rights (GDPR, arts 12–14 and 21, respectively), accord with what we have described as Level 1 consent.

67 An organisation within England comprising the Department of Health and Social Care, NHS England, NHS Digital, and Public Health England, along with representatives from the Information Commissioner’s Office and the National Data Guardian.

68 Information Governance Alliance, ‘The General Data Pr0otection Regulation: Guidance on Consent (Information Governance Alliance, 2018), 3 at <https://digital.nhs.uk/binaries/content/assets/legacy/pdf/2/9/igagdprconsent.pdf> accessed 21 March 2021.

69 ibid.

70 ibid.

71 Further alternatives under art 6 are listed in ibid 7.

72 GDPR, art 9(2)(h). This provision should be read in conjunction with Part 1 of Schedule 1 of the Data Protection 2018.

73 ibid art 9(2)(c).

74 ibid art 9(2)(g). This provision should be read in conjunction with Part 2 of Schedule 1 of the Data Protection 2018.

75 ibid art 9(2)(i). This provision should be read in conjunction with Part 1 of Schedule 1 of the Data Protection 2018.

76 The distinction that may be drawn between a health professional disclosing data for research purposes, and other processing for research purposes (such as that undertaken by the researcher in receipt of the data), is not drawn out in the IGA guidance.

77 Health Research Authority (n 7) above.

78 Data Protection Act 2018, sch 1, para 4.

79 ibid ss 19(2)–(3).

80 For discussion of the requirements of ‘fair’ processing under data protection law, see guidance issued by the Information Commissioner’s Office (ICO): Information Commissioner’s Office, ‘Principle (a): Lawfulness, Fairness and Transparency’ (Guide to the General Data Protection Regulation, May 2019) at <https://ico.org.uk/for-organisations/guide-to-data-protection/guide-to-the-general-data-protection-regulation-gdpr/principles/lawfulness-fairness-and-transparency/#fairness> accessed 21 March 2021. It states, for example, that ‘In general, fairness means that you should only handle personal data in ways that people would expect and not use it in ways that have unjustified adverse effects on them’.

81 General Medical Council (n 30) above, 60–61.

82 There is a logic as to why consent appears to operate differently in the common law and in data protection law. The CLDoC, as the name indicates, emanates from the English common law, and historically, from equity. As jurist Professor Sir Robert Grant Hammond explained in a survey of the duty’s origins, the original remedy for a breach of confidence was by way of injunction, with the Court of Chancery exercising its fundamental jurisdiction to enjoin conduct contrary to conscience (RG Hammond, ‘The Origins of the Equitable Duty of Confidence’ (1979) 8 *Common Law World Review* 71). Data protection law, by contrast, has been instrumentally shaped by developments emanating from Europe, both the Council of Europe and the European Union, arguably even before the first EU-wide law on data protection, the 1995 Data Protection Directive. The European Union’s legislative apparatus understandably has been largely influenced by the civil law regimes that comprise that vast majority of Member States in the EU. (Of the 27 current EU Member States, only Ireland operates within a common law legal system.) The civil law legal system is noted for its reliance on legislative enactments, ie written statutes and other legal codes, drafted by legislators (or civil servants), rather than legal precedents crafted through judicial pronouncement in court cases, the latter of which traditionally have not been considered legally binding.

83 See, eg *X v Y* [1988] 2 All ER 648; *W v Egdell* [1990] Ch 359; *R v Department of Health, ex p Source Informatics* [2000] 1 All ER 786; *Campbell v MGN* (n 26) above; *Vidal-Hall v Google Inc* (n 32) above.

84 As reflected in GDPR, art 7, there are a number of conditions to be met for consent to be valid; but, once those conditions are met, there are fewer surrounding safeguards that attach to the processing of personal data relative to those where a different legal basis operates. We see this, for example, when data are processed in specific situations, such as scientific research. Consent under data protection law puts the onus on individuals—they accept risks they want to accept. The law makes sure that data subjects’ eyes are not closed; they must see (and understand) what they are consenting to. But once a valid consent holds, the data subject is relatively free to set the terms and conditions of the data uses. We also recognise that as per GDPR, art 7(3), consent can be revoked, and revocation must be ‘as easy’ as the giving of consent.

85 It is a principle of data protection law that personal data be processed fairly and lawfully. If there is no valid basis for satisfying the common law duty of confidence, then processing cannot be lawful under data protection law. For a practical application, see the letter from the Office of the Information Commissioner (ICO) to Royal Free NHS Foundation Trust outlining the conclusions of the ICO’s investigation: Elizabeth Denham, ‘RFA0627721 – Provision of Patient Data to DeepMind’ (3 July 2017) at <https://ico.org.uk/media/action-weve-taken/undertakings/2014353/undertaking-cover-letter-revised-04072017-to-first-person.pdf> accessed 21 March 2021.

86 GDPR, art 5(1)(a).

87 This can be justified on public interest grounds: see, eg *W v Egdell* (n 83) above.

88 Let us further consider the circumstances of *W v Egdell* (n 83) above, where a psychiatrist (Dr Egdell) revealed his assessment of a detained mental patient to the authorities to (negatively) influence a decision on moving the patient to a less secure unit. Preparation of assessment was initially with the patient’s consent. When it became clear that the report did not support the patient’s case, the patient declined consent to disclosure of the report to a forthcoming Mental Health Tribunal. While consent to Dr Egdell’s disclosure directly to the tribunal was not technically ‘withdrawn’ (due to the original intent being for disclosure through the solicitors acting for the patient), it is hard to imagine this made any material difference to the Court’s finding that disclosure was in the public interest, lack of consent notwithstanding. If similar facts were to arise today, then the initial processing of sensitive personal data could be on the basis of any one of a number of GDPR art 9 exceptions. Let us imagine that the report is again initially prepared on the basis of the patient’s ‘explicit consent’ (art 9(2)(a)). What we are suggesting is normative alignment between the circumstances in which a court would consider it in the ‘public interest’ to disclose confidential patient information, despite any consent being withdrawn (under the CLDoC), and a move to switch from explicit consent to an alternative exemption under data protection law, eg on the grounds that processing is ‘necessary for reasons of substantial public interest’ (art 9(2)(g)).

89 GDPR, arts 12–14.

90 GDPR, art 21(1).

91 Note that ‘reasonable expectations’ are to be determined by a court and not the patient. When determining whether there is a ‘reasonable expectation of privacy’, the Court of Appeal indicated in *Murray v Express Newspapers plc and another* [2008] EWCA Civ 446 that the question is ‘a broad one, which takes account of all the circumstances of the case. They include the attributes of the claimant, the nature of the activity in which the claimant was engaged, the place at which it was happening, the nature and purpose of the intrusion, the absence of consent and whether it was known or could be inferred, the effect on the claimant and the circumstances in which and the purposes for which the information came into the hands of the publisher’ (Sir Anthony Clarke MR, pp 502–03). Neither the presence or absence of an individual’s consent, nor their objection to processing, is determinative and a court may find use of disclosure of data to be consistent with a reasonable expectation of privacy even where an expressed objection is overridden.

92 GDPR, art 21(6).

93 Our own view is that a Level 1 (opt-out) consent is not a genuine *consent* at all. This aligns with what one of us has written elsewhere: ‘…implied consent is concerned with the scope and interpretation of the signal and does not operate in the absence of a relevant signal. In the absence of any signal at all, the patient’s consent cannot be anything other than imputed. In such circumstances, usage of the language of consent is no more than a fiction’: Taylor, n (9) above, para 12.46 .

94 MJ Taylor and J Wilson, ‘Reasonable Expectations of Privacy and Disclosure of Health Data’ (2019) 27 Medical L Rev 710.

95 *R (W, X, Y and Z)* (n 32) above, [45]–[46]. In this case, certain non-clinical information relating to non-UK resident patients was transmitted by NHS trusts and NHS foundation trusts to the Secretary of State for Health, who then passed it on to the Home Office. The information included the name, date of birth, and gender of the patient, and (if known) his or her current address, the nationality and travel document number with expiry dates, as well as the amount and date of the debt and the NHS body to which it was owed. The claimants were four non-UK residents who were liable to be charged in excess of £1,000 each for NHS services, and were, therefore, liable to immigration sanctions if they failed to pay the charges due. The claimants sought judicial review of the relevant Secretary of State for Health-issued guidance on the National Health Service (Charges to Overseas Visitors) Regulations 2011, SI 2011/1556, on the grounds that, *inter alia*, their disclosed information was private and confidential.

96 ibid para 45 (emphasis added).

97 ibid.

98 Since the GDPR came into force, data protection law has heightened requirements for *any* consent beyond the signal standard. For example, under GDPR, art 7(1), where data processing is based on consent, the controller must be able to demonstrate that the data subject has consented to processing of their personal data. Demonstration of unambiguously indicated behaviour may be difficult in the absence of some recording. Also, under GDPR, art 13(1), data controllers must disclose certain information to data subjects when their data is processed (the so-called ‘privacy notice’), regardless of whether the legal basis for processing is consent. This information includes, among other things: the identity and the contact details of the controller and, where applicable, of the controller’s representative; the contact details of the data protection officer, where applicable; the purposes of the processing for which the personal data are intended as well as the legal basis for the processing; the recipients or categories of recipients of the personal data, if any; and where applicable, whether the controller intends to transfer personal data to a third country or international organisation and if so, the legal basis for doing so. These consent and informational requirements under data protection law undermine the ability to smoothly move from (implied) consent in one regime to another.

99 We have already noted superficial alignment between the explicit consent that professional guidance recommends that health professionals ask for before disclosing identifiable information about patients for purposes other than their care or local clinical audit, and the requirements of an explicit consent under data protection law sufficient for processing personal health data. We do note, however, the specific data protection law requirements for consent and information provision (as a data protection/privacy notice) outlined in particular in GDPR, arts 7 and 13, and the fact that they may impact our claim that the procedural aspect of explicit consent under both the CLDoC and data protection law are substantially similar. This is why we have sought to be clear that we are isolating, for the purposes of this analysis, only the signalling standard.

100 See n 91 above.

101 Information Commissioner’s Office (ICO), ‘Can we change our lawful basis?’ at <https://ico.org.uk/for-organisations/guide-to-data-protection/guide-to-the-general-data-protection-regulation-gdpr/lawful-basis-for-processing/#change> accessed 21 March 2021.

102 See n 85 above.

103 See n 91 above.

104 See n 88 above.

## Notes

The authors would like to thank Graeme Laurie, Roger Brownsword, Lee Bygrave, and Jenny Westaway for their comments on a previous draft. The authors are also grateful to the Academic Research Service at Melbourne Law School for the research assistance provided.

